# Impact of COVID‐19‐related stress on methamphetamine users in Japan

**DOI:** 10.1111/pcn.13220

**Published:** 2021-05-10

**Authors:** Toshihiko Matsumoto, Takashi Usami, Taisuke Yamamoto, Daisuke Funada, Maki Murakami, Kyoji Okita, Takuya Shimane

**Affiliations:** ^1^ Department of Drug Dependence Research, National Institute of Mental Health Tokyo Japan; ^2^ Department of Psychiatry Center Hospital Tokyo Japan; ^3^ Department of Clinical Neuroimaging Integrative Brain Imaging Center, National Center of Neurology and Psychiatry Tokyo Japan

Studies worldwide have observed that COVID‐19 pandemic‐related stress (such as the various constraints of everyday life, and anxiety) promotes the use of drugs as a coping behavior.^1^, ^2^, ^3^, ^4^ Access to alternative maintenance medication of patients with opioid addiction has been reported to have been restricted, resulting in aggravation of their medical conditions^5^; perhaps relatedly, opioid‐overdose deaths have increased.^6^ In Japan, however, the current situation is unknown. We therefore sought to investigate the negative impact of COVID‐19 on drug users who take methamphetamine (MAP), which is one of the most abused drugs in Japan.

Biennially, we conduct national surveys of patients with drug‐related psychiatric disorders who were admitted to or received outpatient treatment at psychiatric hospitals throughout Japan between September and October of that year. Information is collected *via* survey forms mailed to each target hospital, where the attending psychiatrist enters clinical information into the form by transcribing medical records and by conducting interviews. Survey items include demographic variables, education, work status, criminal record, presence/absence of problem drinking, duration of therapy, recovery program (group therapy or self‐help group) participation status, mainly used drug, and ICD‐10 diagnoses. The 2020 survey added two items: COVID‐19‐related relapse (aggravation of substance abuse due to COVID‐19‐related stress, which was clinically determined by each attending psychiatrist), and COVID‐19‐related inhibition of recovery program participation (group therapy and self‐help groups having been temporarily suspended as social distance cannot be ensured in such settings). This study was conducted after approval by the ethics committee of the National Center of Neurology and Psychiatry, and it is conformed to the provisions of the Declaration of Helsinki. The attending psychiatrist gave informed consent, and patient anonymity has been preserved.

In the 2020 survey, 1217 (78.1%) of 1558 target hospitals nationwide provided data on 2733 patients with drug‐related psychiatric disorders (95.6% of 2859 patients consented this survey); of these, the 1461 patients (1076 men, 385 women) who used MAP as a main drug were the subjects of the present study, and 407 patients (27.9%) had used MAP within the past year while the rest (72.1%) had maintained no use of MAP by continuing treatment.

Of the subjects, the 85 patients (5.8%) were considered to have a COVID‐19‐related relapse. Table 1 shows the results of a logistic regression analysis conducted by setting COVID‐19‐related relapse as the dependent variable and the survey terms as independent variables. Male sex (*P* = 0.038, OR 2.031, 95%CI 1.041–3.962), current problem drinking (*P* = 0.012, OR 2.150, 95%CI 1.183–3.905), COVID‐19‐related inhibition of recovery program participation (*P* < 0.001, OR 5.866, 95%CI 3.271–10.520), Dependence Syndrome (*P* < 0.001, OR 7.603, 95%CI 2.569–22.501), and coexisting Childhood/Adolescence‐onset Behavioral and Emotional Disorders (CABED) (*P* = 0.014, OR 2.604, 95%CI 1.213–5.589) were identified as being significantly positively associated with COVID‐19‐related relapse. Duration of therapy ≥1 year (*P* = 0.005, OR 0.416, 95%CI 0.225–0.768) was identified as being significantly negatively associated with relapse.

**Table 1:** Comparison by presence/absence of COVID‐19‐related worsening of drug usage in 1461 cases with methamphetamine‐related disorders

				COVID‐19‐related relapse		Multivariate analysis					
				Yes	No	B	Wald	*P*	Odds ratio	95% CI	
				N=85	N=1376					Lower	Upper
Biological gender (percentage of men)			Frequency	70	1006	0.708	4.316	**0.038**	2.031	1.041	3.962
			%	82.4	73.1						
Age (percentage of subjects ≥40 years old)			Frequency	55	1028	‐0.166	0.379	0.538	0.847	0.500	1.436
			%	64.7	74.7						
Schooling history of ≥12 years			Frequency	50	447	0.438	2.594	0.107	1.549	0.909	2.639
			%	58.8	32.5						
Current employment			Frequency	31	365	‐0.087	0.103	0.748	0.917	0.540	1.557
			%	36.5	26.5						
Custody/arrest record due to drug‐related crimes			Frequency	63	1042	‐0.242	0.585	0.444	0.485	0.422	1.460
			%	74.1	75.7						
Custody/arrest record due to criminal offenses other than drug‐related crimes			Frequency	17	338	‐0.377	1.246	0.264	0.686	0.353	1.330
			%	20.0	24.6						
History of admission to a correctional institution			Frequency	31	772	‐0.440	2.108	0.147	0.644	0.356	1.166
			%	36.5	56.1						
Current problem drinking			Frequency	19	207	0.765	6.314	**0.012**	2.150	1.183	3.905
			%	22.4	15.0						
History of admission to a psychiatric hospital			Frequency	37	847	‐0.224	0.637	0.425	0.799	0.461	1.386
			%	43.5	61.6						
Duration of therapy ≥1 year			Frequency	59	1184	‐0.877	7.860	**0.005**	0.416	0.225	0.768
			%	69.4	86.0						
Participated in a recovery program within the last month			Frequency	48	457	0.001	0.000	0.997	1.001	0.583	1.719
			%	56.5	33.2						
COVID‐19‐related inhibition of participation in recovery programs			Frequency	48	457	1.769	35.243	**<0.001**	5.866	3.271	10.520
			%	56.5	33.2						
ICD‐10 F1 classification subdiagnosis	F1x. 2	Dependence syndrome	Frequency	81	797	2.029	13.429	**<0.001**	7.603	2.569	22.501
			%	95.3	57.9						
	F1x. 5	Psychotic disorder	Frequency	10	200	0.137	0.118	0.731	1.147	0.525	2.506
			%	11.8	14.5						
	F1x. 7	Residual and late‐onset psychotic disorder	Frequency	10	610	‐0.410	1.081	0.299	0.663	0.306	1.438
			%	11.8	44.3						
Comorbid psychiatric disorder	F0	Organic, including symptomatic, mental disorders	Frequency	0	39	‐17.231	0.000	0.998	0.000	–	–
			%	0.0	2.8						
	F2	Schizophrenia, schizotypal and delusional disorders	Frequency	6	154	‐0.150	0.100	0.752	0.860	0.338	2.188
			%	7.1	11.2						
	F3	Mood (affective) disorders	Frequency	17	178	0.341	1.072	0.301	1.406	0.737	2.682
			%	20.0	12.9						
	F4	Neurotic, stress‐related and somatoform disorders	Frequency	12	154	‐0.002	0.000	0.301	0.998	0.485	2.054
			%	14.1	11.2						
	F5	Mental disorders associated with physiological disturbances and physical factors	Frequency	2	22	1.100	1.854	0.173	3.004	0.617	14.632
			%	2.4	1.6						
	F6	Disorders of adult personality and behavior	Frequency	9	82	0.750	3.046	0.081	2.116	0.912	4.911
			%	10.6	6.0						
	F7	Intellectual disabilities (mental retardation)	Frequency	1	60	‐1.037	0.892	0.345	0.355	0.041	3.048
			%	1.6	4.4						
	F8	Disorders of psychological development	Frequency	2	36	‐0.419	0.265	0.607	0.658	0.134	3.048
			%	2.4	2.6						
	F9	Behavioral and emotional disorders with onset usually occurring in childhood and adolescence	Frequency	12	53	0.957	6.027	**0.014**	2.604	1.213	5.589
			%	14.1	3.9						

The results of this study indicate that, for MAP‐dependent patients who recently started their treatment, and therefore, who might not have achieved abstinence yet, the COVID‐related barriers to participation in group therapy and self‐help groups may increase risk of relapse. Our study also suggests that alcohol use (possibly also to cope with COVID‐19‐related stress) may increase the relapse risk in male MAP users. Furthermore, a ‘stay‐at‐home’ lifestyle is speculated to be stressful to promote relapse in those who suffer from attention‐deficit/hyperactive disorder (ADHD), the most common CABED.

These findings may indicate the significance of continuing to implement group therapy and holding self‐help groups under this pandemic, while considering social distance to prevent the spread of infection. This study also suggests that people with drug addictions should be wary when adapting to new ‘self‐restraint’ and ‘stay home’ lifestyles during a pandemic, how to deal with alcohol, the socially accepted substance, and how to reconcile their ADHD, which may be a vulnerable factor for such lifestyle.

This study was conducted in MAP users who had access to a psychiatric hospital. Therefore, there are limitations regarding whether our findings can be generalized to all MAP users. Another limitation is that all study information, including whether the relapse was COVID‐19‐related, was determined solely by each participating hospital's attending psychiatrist. Moreover, the economic impact of the special fixed‐amount benefits (such unexpected income may ironically fund the purchase of MAP promoting relapse), and whether online group therapy and self‐help groups are as effective as in‐person gatherings were not considered.

Despite these limitations, this is the only Japanese study to date to investigate the impact of COVID‐19 on MAP users; therefore this study not only has clinical implications, but is also important with regard to public health.

## Disclosure statement

We declare that all authors have no interest of conflicts.
